# Illness Coherence, Exercise Adherence, and Patient-Reported Outcomes in Chronic Spinal Pain: A Moderated Mediation Analysis

**DOI:** 10.3390/healthcare14172843

**Published:** 2026-09-03

**Authors:** Kalliopi Vlastou, Anastasia Beneka, Evangelos Bebetsos, Maria Basta, Charidimos Tzagarakis, Evangelos Karademas, Izolde Bouloukaki, Dimitris Trivizadakis, Panagiotis Simos

**Affiliations:** 1 Department of Physical Education and Sport Science, Democritus University of Thrace, 69100 Komotini, Greece; k.vlastou@uoc.gr (K.V.); ampeneka@phyed.duth.gr (A.B.); empempet@phyed.duth.gr (E.B.); 2 Psychological and Counseling Support Center, University of Crete, 70013 Heraklion, Greece; 3 School of Medicine, University of Crete, 70013 Heraklion, Greece; haristz@uoc.gr (C.T.); karademas@uoc.gr (E.K.); izolthi@gmail.com (I.B.); simosp@uoc.gr (P.S.); 4 Department of Psychiatry, University Hospital of Heraklion, 71500 Heraklion, Greece; 5 School of Social Sciences, University of Nicosia, Nicosia CY-2417, Cyprus; 6 Directorate of Secondary Education, 71306 Heraklion, Greece; dimcretaergo1@yahoo.gr

**Keywords:** illness coherence, exercise adherence, diaphragmatic breathing, chronic spinal pain, moderated mediation, illness representations, rehabilitation, patient-reported outcomes, quality of life

## Abstract

**Highlights:**

**What are the main findings?**

- Baseline illness coherence was associated with greater unsupervised adherence only in the exercise intervention group.
- Unsupervised exercise adherence mediated the association between illness coherence and patient-reported outcomes, and this indirect association differed according to intervention group.

**What are the implications of the main findings?**

- Illness coherence may be relevant to behavioral engagement in exercise-based rehabilitation for chronic spinal pain.
- The contribution of illness coherence to rehabilitation outcomes may depend on the structure of the intervention.

**Abstract:**

**Background/Objectives**: Illness coherence has been associated with treatment engagement and adherence, but its relationship with exercise adherence and patient-reported outcomes in chronic spinal pain remains unclear. This study examined whether unsupervised exercise adherence mediates the association between baseline illness coherence and patient-reported outcomes and whether this indirect association differs according to the type of intervention received. **Methods**: A theory-driven secondary analysis was conducted using data from a randomized controlled trial of adults with chronic spinal pain receiving either exercise-only or combined exercise plus diaphragmatic breathing intervention (initially randomized = 183; *n* = 57/group). Measures included Revised Illness Perception Questionnaire (IPQ-R), Brief Pain Inventory–Short Form (BPI-SF), Hospital Anxiety and Depression Scale (HADS), World Health Organization Quality of Life Instrument, Brief Version (WHOQOL-BREF), and exercise logs. Moderated mediation analyses examined whether the intervention group moderated the indirect associations between baseline illness coherence and patient-reported outcomes through unsupervised exercise adherence. The parent trial was retrospectively registered at ClinicalTrials.gov (NCT07539116) on 12 April 2026. **Results**: Mean age was 46.95 ± 14.54 and 46.32 ± 15.97 years, and women comprised 40.4% and 47.4% of the exercise-only and combined groups, respectively. Baseline illness coherence was positively associated with unsupervised exercise adherence in the exercise-only group (b = 71.04, *p* = 0.018). Significant conditional indirect effects of illness coherence on all patient-reported outcomes through unsupervised exercise adherence were observed in the exercise-only group (b = −1.55 to 1.32, ps < 0.05), but not in the combined intervention group (b = −0.59 to 0.32, ps > 0.05). Indices of moderated mediation were significant across all outcomes (all ps < 0.05). No intervention-related adverse events were reported. **Conclusions**: Diaphragmatic breathing may reduce the relative contribution of illness-related cognitive representations on patient-reported outcomes by introducing additional self-regulatory pathways through which clinical improvement is achieved.

## 1. Introduction

Chronic spinal pain is a major contributor to disability and represents a substantial public health challenge because of its high prevalence, recurrent course, and pervasive effects on physical, psychological, and social functioning [1,2]. Contemporary models increasingly view chronic spinal pain as a multifactorial condition involving biological as well as psychological and behavioral factors that influence symptom experience, coping, and adaptation [2]. Consequently, current clinical guidelines advocate a biopsychosocial approach to management, placing particular emphasis on non-pharmacological interventions and especially on exercise-based rehabilitation as a cornerstone of long-term care [1], supported by systematic evidence on the efficacy of exercise therapy [3].

Exercise interventions have consistently demonstrated beneficial effects on pain intensity, physical functioning, and quality of life; however, their effectiveness depends not only on the characteristics of the intervention itself but also on patients’ sustained engagement with the prescribed activities [3,4,5]. In this context, exercise adherence, particularly adherence to unsupervised home-based exercise, has emerged as a critical determinant of rehabilitation success. Nevertheless, maintaining regular exercise behavior over time remains challenging for many individuals with chronic spinal pain, suggesting that clinical and demographic factors alone are insufficient to explain variability in rehabilitation outcomes [4,5,6,7]. Notably, the initiation of home-based exercise appears to be conceptually and behaviorally distinct from its long-term maintenance, with evidence for interventions specifically targeting long-term adherence remaining limited [8]. A recent umbrella review of 19 systematic reviews and 205 trials further found only small but significant effects of behavior change techniques, booster sessions, and goal-setting interventions on adherence [9].

Increasing attention has therefore been directed toward psychological factors that may shape treatment engagement and self-management. Evidence regarding the psychological determinants of exercise adherence, in particular, remains heterogeneous, with studies emphasizing different explanatory mechanisms, including illness beliefs, self-efficacy, social support, pain severity, and intervention-related characteristics [5,6,7,10,11,12]. Social support in particular has recently been shown to significantly predict exercise adherence following discharge from treatment in patients with chronic low back pain, independent of self-efficacy [13]. Qualitative and quantitative evidence indicates that individuals who perceive rehabilitation activities as meaningful and congruent with their understanding of the condition are more likely to sustain home-based exercise over time, whereas uncertainty, conflicting beliefs, and limited understanding may undermine adherence [6,7,11,12].

Among psychological factors that may shape treatment adherence, patients’ representations of their illness appear particularly relevant. According to the Common-Sense Model of Self-Regulation (CSM), individuals construct cognitive and emotional representations of their illness, which guide coping responses and are continuously revised in light of symptom experience and treatment outcomes [14,15]. Illness coherence is a central component of patients’ illness representations, as it reflects the extent to which they perceive their condition as understandable and meaningful [16,17]. Greater illness coherence has been associated with more adaptive self-regulatory processes, greater engagement in health-promoting behaviors, and more favorable clinical outcomes across a wide range of chronic conditions [10,14,15,18,19]. A recent multilevel meta-analysis of 93 studies (N = 18,262) confirmed that illness coherence is positively associated with perceived control and negatively associated with perceived illness threat [19]. Conversely, limited understanding of one’s condition may hinder effective coping, increase uncertainty about treatment recommendations, and reduce the likelihood of sustained participation in self-management behaviors [10,18].

Despite growing recognition of the importance of illness coherence, relatively little is known about the mechanisms through which it may influence rehabilitation outcomes in chronic spinal pain [10,16,18]. One plausible pathway involves exercise adherence. Exercise is widely recommended as a non-pharmacological treatment for chronic spinal pain, and adherence to prescribed exercise is associated with improved clinical outcomes [1,3,4,5,6,7]. Therefore, identifying factors that promote exercise adherence is particularly important in this population. However, the role of specific behavioral indicators, particularly unsupervised exercise adherence, has received comparatively little attention. In multimodal rehabilitation programs, additional components such as diaphragmatic breathing may also target physiological and emotional regulation [20,21]. Whether the indirect pathway linking illness coherence, exercise adherence, and patient-reported outcomes differs according to intervention type remains unclear.

The present study aimed to examine whether unsupervised exercise adherence mediates the association between baseline illness coherence and patient-reported outcomes (pain intensity, anxiety and quality of life indicators). It was hypothesized that greater illness coherence would be associated with better outcomes through increased exercise adherence. A secondary aim was to examine whether the magnitude of this indirect association depends upon the type of intervention administered. Specifically, it was postulated that systematic training on relaxation techniques, such as diaphragmatic breathing, may reduce the dependence of positive outcomes on pre-existing illness representations. Consistent with this hypothesis, findings indicated that illness coherence was associated with more favorable patient-reported outcomes through greater unsupervised exercise adherence, but only among participants receiving exercise alone, suggesting that the addition of diaphragmatic breathing may attenuate this cognitive-behavioral pathway.

## 2. Materials and Methods

### 2.1. Study Design

Data were drawn from a three-arm parallel-group, superiority randomized controlled trial designed to investigate the effects of a combined exercise plus diaphragmatic breathing intervention on pain-related, psychological, cognitive, and quality-of-life outcomes in individuals with chronic non-specific spinal pain. Participants were randomly assigned in a 1:1:1 ratio to one of three groups: (a) a combined exercise plus diaphragmatic breathing group, (b) an exercise group, or (c) a minimal intervention control group who received general health guidance without active intervention. The present study reports a theory-driven secondary analysis restricted to the two active intervention arms. Participants assigned to the minimal intervention control group were not included in the moderated mediation analyses because exercise adherence constituted the mediator of interest and was therefore not applicable to the control condition. Primary outcomes of the parent trial are reported separately (manuscript under review, Behavioral Medicine, which does not include any of the present analyses). Patients and the public were not involved in the design, conduct, or reporting of this study.

### 2.2. Procedure

Participants were recruited between October 2024 and May 2025 through an open invitation disseminated across various professional and clinical settings in Heraklion, Crete, Greece. Recruitment announcements included a brief description of the study objectives, intervention procedures, duration of participation, and basic eligibility criteria. Invitations were distributed to employees of the University of Crete, employees of the University Hospital of Heraklion, and individuals receiving care from collaborating private orthopedic practices.

Individuals who expressed interest in participating underwent an initial screening procedure to determine their eligibility according to the inclusion criteria. The screening included the collection of basic demographic and clinical information, as well as a brief assessment of symptom duration and pain characteristics. Eligible individuals received detailed information regarding the study aims, procedures, potential benefits, and possible inconveniences associated with participation and were informed of their right to withdraw from the study at any time without consequences. Written informed consent was obtained from all participants prior to enrollment.

Following written informed consent, they underwent baseline assessment (T1), during which demographic and clinical data were collected, and all study measures were administered. Baseline assessment was completed before randomization to ensure that the initial measurements were not influenced by knowledge of group allocation. Recruitment and enrollment were conducted in four consecutive waves, beginning in October 2024 and continuing in January 2025, March 2025, and May 2025. A total of 183 participants meeting the eligibility criteria were enrolled and randomized to one of the three study groups.

The trial was retrospectively registered at ClinicalTrials.gov (NCT07539116; registered 12 April 2026; https://clinicaltrials.gov/study/NCT07539116, accessed on 15 June 2026). Retrospective registration occurred because the trial was initially considered methodologically linked to a separate prospectively registered study (NCT06666478) conducted by the same research group using the same core exercise protocol. Once it was recognized that the present trial constituted an independent study, separate registration was completed.

The study design, participant flow, and trial reporting were in accordance with the CONSORT (Consolidated Standards of Reporting Trials) recommendations for randomized controlled trials [22] (see  Supplementary Figure S1).

The randomization sequence was generated by the lead researcher (K.V.), a doctoral candidate conducting the study, before recruitment began, using a computer-generated block sequence (Research Randomizer, Version 4.0, Social Psychology Network) with variable block sizes (6 and 9). Allocation concealment was ensured through sequentially numbered, opaque, sealed envelopes prepared in advance and opened only after baseline assessment and eligibility confirmation, in strict numerical order. The exercise professional delivering the interventions was not involved in randomization and was informed of group allocation only after baseline assessment.

Full blinding of participants and intervention providers was not feasible given the nature of the interventions. To limit potential bias, statistical analyses were conducted by an independent analyst using an anonymized dataset with a coded group variable, interventions were delivered under standardized protocols by the same exercise professional, and cross-contamination between groups was minimized by avoiding joint sessions or shared educational material across intervention arms.

**Figure 1:**
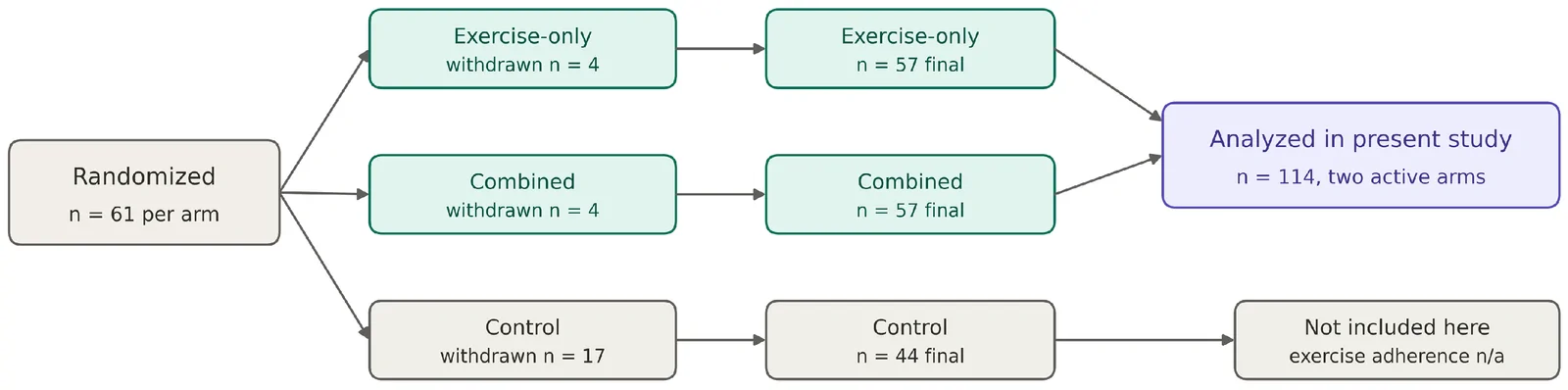
Participant flow from randomization to the analytic sample of the present secondary analysis. Withdrawals occurred primarily due to participants initiating a parallel therapeutic intervention for the same condition. The control arm was not included in the present analysis because participants did not receive an active exercise intervention, making exercise adherence not applicable as a mediator for this arm.

Of the 183 participants randomized (*n* = 61 per group), 158 completed the parent trial; withdrawals were primarily due to participants starting a parallel therapeutic intervention for the same condition during the first two weeks. Discontinuation due to initiation of parallel treatment was markedly more frequent in the control arm (*n* = 17, 27.9%) than in either active intervention arm (*n* = 4, 6.6% in each). Control participants received only general written and verbal health guidance (walking, stretching, and neuromuscular relaxation) rather than therapist-supervised intervention, which may have contributed to their greater likelihood of seeking treatment elsewhere (Figure 1).

### 2.3. Participants

Eligible participants were 114 adults (50 women) aged between 18 and 72 years with a medically confirmed diagnosis of chronic non-specific spinal musculoskeletal pain affecting the cervical, thoracic, or lumbar spine. Chronic pain was defined as pain persisting for at least 12 weeks or as recurrent pain episodes with temporal persistence. Additional inclusion criteria were medical clearance for safe participation in the study interventions, no use of opioid analgesics during the month preceding enrollment, no participation in another structured physical or psychological intervention for spinal pain before enrollment, and sufficient understanding of the study procedures to provide written informed consent.

Individuals were excluded if they presented with acute spinal pathology (e.g., recent injury or active inflammation), neurological or severe neuromuscular disorders, clinically significant psychiatric disorders, active substance dependence, chronic respiratory disease, pregnancy, or any medical condition that rendered participation in the interventions unsafe. Participants were also excluded if they had insufficient knowledge of the Greek language to understand the study procedures and questionnaires or if they had participated in another therapeutic intervention for spinal pain during the month preceding enrollment or at the time of enrollment.

### 2.4. Interventions

Participants in the combined exercise plus diaphragmatic breathing group received a standardized exercise program combined with diaphragmatic breathing delivered through a biofeedback-assisted protocol. At baseline, resting breathing rate was assessed, and participants were trained in slow-paced diaphragmatic breathing targeting 6–8 breaths/min using nasal inhalation and prolonged exhalation [20]. Respiratory and cardiovascular activity were monitored using a biofeedback system (Nexus 10, Mind Media BV, Roermond, The Netherlands) equipped with respiratory and blood volume pulse sensors. Weekly supervised sessions consisted of 20 min of diaphragmatic breathing training and 40 min of exercise. In addition, participants performed four unsupervised breathing sessions per week (10 min/session) and two home-based exercise sessions, corresponding to approximately 100 min of exercise and 60 min of breathing practice per week.

Participants in the exercise group followed the same standardized exercise program targeting mobility, muscle strengthening, postural control, flexibility, and neuromuscular coordination through functional body-weight exercises. The protocol consisted of 18 standardized seated and standing exercises involving the spine and extremities, emphasizing controlled movement execution, proper alignment, and breathing coordination, with 30 s of exercise followed by 20 s of rest. Participants attended four initial instructional sessions followed by one supervised session per week throughout the 12-week intervention period. In addition, they completed two home-based exercise sessions per week of approximately 30 min each, resulting in a total exercise volume of approximately 100 min per week. Written instructions were provided to facilitate home practice. All supervised exercise sessions were delivered by the same qualified exercise professional, whereas diaphragmatic breathing training and psychoeducation were delivered by the principal investigator. Adherence was monitored using weekly self-report logs documenting the frequency, type, and duration of home-based exercise and breathing practice. No intervention-related adverse events were reported.

### 2.5. Measures

Measures relevant to the present analysis included pain intensity and pain interference, cognitive flexibility, anxiety, and depressive symptoms. Secondary outcomes comprised quality of life and adherence to the prescribed home exercise program. Additional cognitive and psychosocial measures included non-verbal intelligence and inductive reasoning, illness perceptions, and coping strategies. Pain intensity and pain interference were assessed using the Brief Pain Inventory–Short Form (BPI-SF) [23,24]. Anxiety and depressive symptoms were assessed using the Hospital Anxiety and Depression Scale (HADS) [25]. Quality of life was assessed using the World Health Organization Quality of Life instrument, short form (WHOQOL-BREF) [26]. Illness coherence was assessed using the corresponding scale from the Revised Illness Perception Questionnaire (IPQ-R) [16]. Exercise adherence was operationally defined as the total duration of home-based exercise recorded through weekly self-report logs, and was also expressed as a percentage of the prescribed weekly home-exercise dose (two 30-min sessions per week over the 12-week intervention; total prescribed dose = 720 min), to facilitate interpretation across participants. Validated Greek-language versions were used where available. Assessments were conducted at three time points: baseline prior to randomization (T1), immediately after completion of the 12-week intervention period (T2), and at a one-month follow-up (T3).

### 2.6. Statistical Analysis

The present secondary analysis used moderated mediation models to examine potential behavioral pathways linking baseline illness coherence to subsequent patient-reported outcomes. For this analysis, illness coherence assessed at T1 was specified as the predictor, exercise adherence, aggregated as a continuous measure over the intervention period (T1 to T2), as the mediator, and intervention group as the moderator (Group 1: Exercise only, Group 2: combined exercise plus diaphragmatic breathing intervention). Outcome measures assessed at T3 included pain intensity, pain interference with daily functioning, anxiety symptoms, and the physical and psychological dimensions of quality of life. Gender, age, and years of education were included as covariates in all analyses. There were no missing data in the analytic dataset. Moderated mediation analyses were performed using the PROCESS macro for SPSS (version 3.0), specifically Model 7, which estimates conditional indirect effects when the association between the independent variable and the mediator is moderated by a third variable [27]. Because the index of moderated mediation constituted the primary test of whether the indirect pathway differed between intervention groups, Bonferroni correction was applied across the five indices of moderated mediation (α = 0.01). Conditional indirect effects were interpreted at the conventional α = 0.05 level as follow-up estimates of the indirect pathway within each intervention group. Anxiety and pain interference at T1 were additionally entered as covariates in supplementary sensitivity models. All analyses were conducted using IBM SPSS Statistics version 23.0 (IBM Corp., Armonk, NY, USA).

## 3. Results

### 3.1. Baseline Characteristics

A total of 114 participants were included in the secondary analysis, comprising 57 participants in the exercise intervention group and 57 participants in the combined exercise plus diaphragmatic breathing intervention group. Baseline demographic, clinical, psychological, and cognitive characteristics are presented in Table 1.

The mean age of the participants was 46.95 ± 14.54 years in the exercise-only intervention group and 46.32 ± 15.97 years in the combined exercise plus diaphragmatic breathing intervention group. Women represented 40.4% and 47.4% of participants in the two groups, respectively. Overall, the two groups were comparable across most demographic, clinical, and psychological variables at baseline, with two notable exceptions. On average, the exercise-only group reported higher anxiety symptoms (*p* = 0.026), yet they reported lower pain interference (*p* = 0.034). Bivariate correlations among the study measures in the total sample are presented in Table 2. Percentage adherence to the prescribed home exercise dose averaged 75.3% (SD = 19.8%) across the analytic sample (exercise-only: M = 64.1%, SD = 17.5%; combined: M = 86.5%, SD = 15.1%).

### 3.2. Moderated Mediation Analysis

The moderated mediation model tested whether the indirect effect of baseline illness coherence on patient-reported outcomes, through unsupervised exercise adherence, differed according to intervention group (exercise-only vs. combined); the full path diagram is presented in Supplementary Figure S2. Across the five outcome models, the unmoderated association between baseline illness coherence and unsupervised exercise adherence was not significant (b = 19.60, *p* = 0.319, 95% CI [−19.22, 58.42]).

However, across both intervention groups, home exercise adherence was positively associated with physical quality of life (b = 0.02, *p* < 0.001, 95% CI [0.01, 0.02]), psychological quality of life (b = 0.02, *p* < 0.001, 95% CI [0.01, 0.02]), pain intensity (b = −0.01, *p* < 0.001, 95% CI [−0.012, −0.008]), pain-related interference (b = −0.01, *p* < 0.001, 95% CI [−0.012, −0.008]), and anxiety symptoms (b = −0.01, *p* < 0.001, 95% CI [−0.012, −0.008]), after adjustment for illness coherence and the covariates.

#### 3.2.1. Moderation of the Association Between Illness Coherence and Exercise Adherence

The association between illness coherence and exercise adherence differed between groups as indicated by a significant interaction effect, *b* = −102.88, *p* = 0.008, 95% CI [−178.93, −26.82]. Inspection of conditional effects indicated that illness coherence was positively and significantly associated with exercise adherence in the exercise-only intervention group (Group 1), *b* = 71.04, *p* = 0.018, 95% CI [12.32, 129.77]. In contrast, the association did not reach significance in the combined exercise plus diaphragmatic breathing intervention group (Group 2): *b* = −31.84, *p* = 0.206. A conventional post-hoc power estimate based on the observed interaction between illness coherence and intervention group in the mediator model (*b* = −102.88, SE = 38.37, *t* = −2.68, *p* = 0.008) indicated approximately 76% power to detect this moderation effect at α = 0.05.

These findings suggest that illness coherence functioned as a significant correlate of exercise adherence only when exercise constituted the primary behavioral component of the intervention. In contrast, when diaphragmatic breathing was introduced as an additional active component, the relationship between illness coherence and adherence to home-based exercise was no longer evident. This pattern justified the subsequent examination of conditional indirect effects separately for each intervention group.

#### 3.2.2. Moderation of the Indirect Effect of Illness Coherence on the Study Outcomes

At the conventional 95% confidence level, significant conditional indirect effects of illness coherence on each of the five study outcomes, through exercise adherence, were restricted to the exercise-only intervention group. These effects were positive for physical (effect = 1.32, bootstrapped 95% CI [0.13, 2.84]) and psychological quality of life (effect = 1.17, bootstrapped 95% CI [0.11, 2.52]), and negative for pain intensity (effect = −0.70, bootstrapped 95% CI [−1.50, −0.06]), pain interference (effect = −0.72, bootstrapped 95% CI [−1.51, −0.07]), and anxiety symptoms (effect = −0.71, bootstrapped 95% CI [−1.55, −0.07]). Adjusting for baseline pain interference and anxiety symptoms yielded a highly similar pattern of conditional indirect effects: physical (effect = 1.29, bootstrapped 95% CI [0.24, 2.62]) and psychological quality of life (effect = 1.16, bootstrapped 95% CI [0.25, 2.43]), and negative for pain intensity (effect = −0.71, bootstrapped 95% CI [−1.40, −0.14]), pain interference (effect = −0.72, bootstrapped 95% CI [−1.42, −0.18]), and anxiety symptoms (effect = −0.70, bootstrapped 95% CI [−1.39, −0.14]). These conditional indirect effects did not meet the more stringent Bonferroni-adjusted α = 0.01 criterion. Corresponding conditional indirect effects failed to reach significance in the combined intervention group. The index of moderated mediation indicated significant differences in the indirect effect between intervention groups for Psychological Quality of Life: index = −1.70, 99% CI [−3.95, −0.06], pain interference: index = 1.05, 99% CI [0.03, 2.33], and anxiety symptoms: index = 1.03, 99% CI [0.01, 2.37], based on the Bonferroni-adjusted 99% confidence intervals. For the remaining outcomes, the index of moderated mediation was significant at the conventional α = 0.05 level but did not meet the adjusted α = 0.01 criterion (Physical Quality of Life: index = −1.91, 95% CI [−3.66, −0.45] and pain intensity: index = 1.02, 95% CI [0.26, 1.92]). Corresponding indices after controlling for pain interference and anxiety at T1 were as follows: Physical Quality of Life: index = −1.64, 99% CI [−3.94, −0.04], Psychological Quality of Life: index = −1.47, 99% CI [−3.50, −0.01], pain intensity: index = 0.90, 99% CI [0.05, 2.00], pain interference: index = 0.92, 99% CI [0.03, 2.06], anxiety symptoms: index = 0.89, 99% CI [0.36, 2.05].

The moderated mediation model illustrating the conditional indirect effects by intervention group is presented in Supplementary Figure S2. Although physical (pain-related and physical well-being) and anxiety-related symptoms were the primary target of the present study, in view of the nature of the intervention employed, a final exploratory model was conducted, using depression symptoms at T3 as the outcome. Again, the index of moderated mediation was significant (index = 1.00, 99% CI [0.05, 2.30]) even after controlling for age, gender, education, anxiety symptoms and pain interference at T1, and the significant indirect effect of illness coherence through exercise adherence was restricted to the exercise-only intervention group (effect = −0.79, bootstrapped 99% CI [−1.81, −0.02]).

## 4. Discussion

The present study examined whether illness coherence contributes to patient-reported outcomes by influencing exercise adherence, and whether this pathway differs by intervention modality. The findings indicate that illness coherence was associated with more favorable patient-reported outcomes through increased engagement in unsupervised exercise, but only among participants assigned to the exercise-only intervention. In contrast, exercise adherence did not mediate the relationship between illness coherence and outcomes among participants assigned to the combined exercise and diaphragmatic breathing intervention. These findings suggest that illness coherence and exercise adherence may represent important behavioral pathways through which clinical improvement may occur when exercise constitutes the primary therapeutic strategy; additional self-regulatory interventions, such as diaphragmatic breathing, may reduce the relative contribution of this pathway [10,14,15,20,21].

The first main finding of this study was that baseline illness coherence was positively associated with unsupervised exercise adherence in the exercise-only intervention group, while this association did not reach statistical significance in the combined exercise plus diaphragmatic breathing intervention group. The observed pattern is consistent with the Common-Sense Model (CSM) of self-regulation, which proposes that individuals develop cognitive representations of their illness that guide coping behavior and influence subsequent health outcomes [10,14,15]. Within this framework, illness coherence reflects the extent to which individuals perceive their condition as understandable and make sense of their symptoms and illness-related experiences [14,15,16]. Individuals with a clearer understanding of their condition are more likely to engage in health-promoting behaviors, adhere to treatment recommendations, and sustain self-management strategies over time [10,18]. These findings suggest that coherent illness representations may be particularly important when patients are expected to rely primarily on their own behavioral engagement to achieve therapeutic benefits.

The second main finding of this study was that the mediating role of exercise adherence was consistent across all outcomes examined. Higher illness coherence predicted greater engagement in unsupervised exercise, which in turn was associated with better physical and psychological quality of life, lower pain intensity, reduced pain interference, and lower anxiety and depression symptoms. Such convergence suggests that exercise adherence may operate as a common pathway linking cognitive illness representations to a broad range of clinically meaningful outcomes. This interpretation is compatible with contemporary biopsychosocial models of chronic pain, which emphasize the reciprocal interplay among cognitive, behavioral, emotional, and physiological processes rather than peripheral nociceptive mechanisms alone [2]. Our findings are consistent with previous reports that adherence to exercise interventions is a key determinant of successful rehabilitation in chronic musculoskeletal pain [4,5,6,7,11,12]. Exercise adherence may therefore be viewed not merely as a behavioral outcome but as one of the principal behavioral routes through which these cognitive processes are translated into longer-term adaptation [10,14,15].

The third main finding of this study, which extends previous research on illness perceptions and exercise adherence, was that the indirect pathway did not reach statistical significance in the combined intervention condition. Participants receiving exercise and diaphragmatic breathing achieved favorable outcomes in the parent trial, yet neither the association between illness coherence and exercise adherence nor the indirect effect through adherence reached statistical significance. This non-significant finding should be interpreted with caution, as it may reflect insufficient statistical power to detect the hypothesized effect within this subgroup (*n* = 57) rather than the true absence of an underlying association (see Limitations). This divergence suggests that the addition of diaphragmatic breathing may have altered the self-regulatory processes supporting clinical improvement. Different intervention components may engage partially distinct self-regulatory processes, thereby reducing the relative contribution of any single behavioral pathway to treatment response. According to the Common-Sense Model, individuals select coping strategies that align with their understanding of the illness and their perceptions of treatment effectiveness [14,15]. When exercise is the sole active component of treatment, engagement in exercise is likely to represent the primary means through which participants translate illness-related beliefs into adaptive action; diaphragmatic breathing, by contrast, may provide an additional self-regulatory strategy for symptom management, while reductions in physiological stress responses and autonomic arousal may contribute directly to clinical improvement [20,21]. In this context, therapeutic benefits may have been distributed across multiple behavioral and physiological pathways rather than depending primarily on exercise engagement.

It is important to stress that the present study was not designed to throw light on the processes responsible for the attenuated indirect effect of illness coherence, given the lack of measures of autonomic regulation, breathing adherence, self-regulatory capacity, and physiological stress responses. Two testable accounts merit consideration in future studies. First, diaphragmatic breathing may have acted as a primary, bottom-up regulator of distress, operating relatively independently of patients’ cognitive representations of their illness. This account is consistent with the respiratory vagal stimulation model, which proposes that voluntarily slowed breathing directly enhances vagally mediated parasympathetic activity and thereby supports emotional regulation and stress recovery [28]. Meta-analytic evidence confirms that voluntary slow breathing reliably increases vagally mediated heart rate variability [20], and conclusions from a systematic-review evidence links slow breathing techniques to reduced physiological arousal and increased subjective relaxation [29]. Experimental work further indicates that deep slow breathing can attenuate pain perception and sympathetic arousal, although these effects appear to depend on the relaxing quality of the breathing rather than on respiratory rate alone [21]. Furthermore, a systematic review of respiration–pain interactions concluded that the mechanisms underlying such hypoalgesic effects remain incompletely understood [30]. Consistent with the possibility of a direct, adherence-independent route to symptom improvement, recent meta-analyses of randomized controlled trials in chronic low back pain indicate that breathing exercises by themselves reduce pain intensity and disability [31,32] and may additionally modify cognitive–affective processes such as fear-avoidance beliefs [32], although the certainty of this evidence has been rated as low. Within the Common-Sense Model, coping procedures are selected in accordance with patients’ illness representations [14,15]; a readily available, bottom-up regulatory strategy of this kind could weaken the dependence of adaptive coping—and of subsequent outcomes—on a coherent cognitive understanding of the condition, so that participants with lower illness coherence could nonetheless benefit, thereby diluting the adherence-mediated pathway observed when exercise was the sole active component. Second, a floor-effect account cannot be discounted: if the addition of breathing training produced earlier or broader symptom relief, the resulting restriction in residual symptom severity may have limited the variance available for the illness coherence–adherence–outcome pathway.

From a clinical perspective, the findings highlight the importance of considering illness coherence and exercise adherence as modifiable treatment targets in rehabilitation programs for chronic spinal pain. Interventions aimed at improving patients’ understanding of their condition, strengthening self-management skills, and enhancing confidence in therapeutic strategies may facilitate greater adherence to prescribed exercise and, consequently, improve long-term outcomes [4,5,6,7,10,14,15]. At the same time, the present findings suggest that interventions incorporating diaphragmatic breathing may offer benefits through mechanisms that extend beyond behavioral adherence alone. Such interventions may therefore be particularly useful for individuals who experience difficulties maintaining exercise routines or who present with substantial emotional distress and stress-related symptom exacerbation.

Several strengths of the present study should be acknowledged. The analysis was based on data derived from a randomized controlled trial and examined multiple clinically relevant patient-reported outcomes, including pain, quality of life, and anxiety and depression symptoms. A further strength is the interventional and longitudinal structure of the parent randomized controlled trial, which allowed baseline illness coherence, exercise adherence during the intervention period, and patient-reported outcomes at follow-up to be examined in a temporally ordered framework. In addition, the use of moderated mediation analyses allowed a more nuanced understanding of the behavioral and cognitive pathways underlying treatment outcomes and provided insight into how different intervention modalities may operate through distinct mechanisms of change.

Nevertheless, certain limitations should also be considered. First, the present investigation represents a secondary analysis and was not designed to ensure adequate power for moderated mediation analyses. A conventional post-hoc estimate based on the observed illness coherence × intervention group interaction indicated approximately 76% power at α = 0.05. Because this estimate pertains to the moderation component rather than the bootstrap-based conditional indirect effect itself, the moderated mediation findings should be interpreted cautiously. Second, exercise adherence was assessed exclusively through self-reported unsupervised exercise time, which may be subject to recall bias and social desirability bias, potentially resulting in systematic overestimation of actual adherence levels. Adherence was recorded prospectively through structured weekly logs, supported by regular researcher contact and reminders, rather than through a single retrospective report at the end of the intervention period. Furthermore, self-reported unsupervised exercise duration was significantly correlated with objectively recorded attendance at supervised sessions, both in terms of total minutes (r = 0.58, *p* < 0.001) and number of sessions (r = 0.67, *p* < 0.001), providing convergent evidence that self-reported adherence was not disconnected from independently documented engagement with the intervention. Nevertheless, because participants were aware that adherence constituted a central focus of the study, socially desirable responding may have persisted despite these safeguards, and this convergent association does not rule out a uniform overestimation bias across participants. Such overestimation, if differential across participants, could have inflated or attenuated the observed associations between illness coherence, adherence, and outcomes; however, the direction and magnitude of any resulting bias cannot be determined with the available data. Third, the study did not include objective physiological indicators of autonomic regulation or direct measures of diaphragmatic breathing practice, preventing definitive conclusions regarding the mechanisms underlying the combined intervention. Fourth, modest baseline differences in anxiety symptoms and pain interference were observed despite randomization. Although these differences likely reflect chance variation and sensitivity analyses indicated that they did not influence the overall patterns of results, they should be considered when interpreting the findings. Fifth, the parent trial was registered retrospectively, after recruitment had already commenced. As explained in the Materials and Methods section, this occurred due to an initial misunderstanding regarding the registration requirements, given the methodological overlap with a separate, prospectively registered trial conducted by the same research group. Nonetheless, retrospective registration remains a recognized methodological limitation, as it increases the potential for undisclosed post hoc changes to the outcomes, hypotheses, or analytic approach relative to what may have been originally intended. Sixth, the high correlations among some patient-reported outcomes, particularly pain intensity and interference and anxiety and depression symptoms, raise the possibility of measurement overlap and common-method/reporting effects, whereby a general negative symptom appraisal may have limited participants’ differentiation among related symptom domains. This may have contributed to shared variance and inflated associations among some outcomes, consistent with previously reported associations between BPI severity and interference subscales in non-cancer pain populations [33]. Seventh, baseline anxiety and pain interference were entered as covariates in supplementary sensitivity analyses without altering the pattern of results. Eighth, the individual conditional indirect effects did not consistently meet the Bonferroni-adjusted significance threshold, which may partly reflect the modest sample size and the reduced power associated with multiple testing. Finally, although moderated mediation analyses provide valuable information regarding potential pathways of association, causal inferences regarding the underlying mechanisms should be made cautiously.

The present findings have potential implications for rehabilitation planning in chronic spinal pain. Because illness coherence was associated with exercise adherence only when rehabilitation relied primarily on exercise, clinicians may consider paying greater attention to patients’ understanding of their condition when prescribing long-term home-based exercise programs. In this context, structured patient education or pain neuroscience education aimed at improving illness understanding, explaining the therapeutic rationale for exercise, addressing maladaptive beliefs, and strengthening self-management skills may facilitate engagement with home-based rehabilitation [1,34,35,36]. This approach is consistent with current recommendations emphasizing education, supported self-management, exercise, and person-centered biopsychosocial care in chronic low back pain management [1,34,35]. By contrast, when rehabilitation includes additional stress-regulation interventions, such as diaphragmatic breathing, clinical improvement may rely less on exercise adherence alone and more on complementary behavioral and physiological mechanisms [20,21]. Although these findings require prospective confirmation, they support considering illness coherence during routine rehabilitation planning to guide the intensity of educational support and adherence-promoting strategies rather than to determine treatment allocation.

Future research should investigate the physiological and psychological mechanisms through which combined exercise and diaphragmatic breathing interventions influence adaptation to chronic spinal pain. The inclusion of objective measures of autonomic regulation, such as heart rate variability, as well as more detailed assessments of breathing adherence and self-regulatory processes, may provide a clearer understanding of the pathways involved [20]. Furthermore, longitudinal studies with longer follow-up periods are needed to determine whether the observed relationships between illness coherence, exercise adherence, and patient-reported outcomes are maintained over time and whether different intervention components exert distinct long-term effects on adaptation to chronic pain.

## 5. Conclusions

The present study provides evidence consistent with the hypothesis that illness coherence and exercise adherence may represent important behavioral pathways linking cognitive illness representations to patient-reported outcomes in individuals with chronic spinal pain. Specifically, illness coherence was associated with improved physical and psychological outcomes through increased exercise adherence only when exercise constituted the primary behavioral component of the intervention. In contrast, this indirect pathway did not reach statistical significance in the combined exercise plus diaphragmatic breathing intervention, suggesting that additional mechanisms of change may operate when multiple therapeutic components are introduced. As illness coherence was not experimentally manipulated in this secondary analysis, these findings should be interpreted as hypothesis-generating rather than as direct evidence that modifying illness coherence would improve adherence or outcomes. Nonetheless, they highlight the potential relevance of cognitive illness representations and behavioral adherence processes in the design of rehabilitation programs for chronic spinal pain and support further, ideally experimental, investigation of the mechanisms through which different intervention strategies influence long-term adaptation to chronic pain.

## Figures and Tables

**Table 1:** Baseline demographic, clinical, psychological and cognitive characteristics of participants included in the secondary analysis.

Variable	Exercise-Only Intervention	Combined Exercise Plus Diaphragmatic Breathing Intervention	*p* Value
Demographic characteristics			
Age, years	46.95 ± 14.54	46.32 ± 15.97	0.826
Women, *n* (%)	23 (40.4)	27 (47.4)	0.571
Years of education	13.19 ± 3.08	12.35 ± 3.38	0.168
BMI category, *n* (%)			0.717
Normal weight	13 (22.8)	17 (29.8)	
Overweight	29 (50.9)	23 (40.4)	
Obesity I	8 (14.0)	9 (15.8)	
Obesity II	7 (12.3)	8 (14.0)	
Clinical characteristics			
Neck pain, *n* (%)	41 (71.9)	43 (75.4)	0.832
Low back pain, *n* (%)	56 (98.2)	57 (100.0)	1.00
Thoracic pain, *n* (%)	48 (84.2)	37 (64.9)	0.030
Pain intensity (BPI-SF, scale 0–10)	8.07 ± 0.62	8.12 ± 0.74	0.697
Pain interference (BPI-SF, scale 0–10)	7.34 ± 0.79	7.68 ± 0.90	0.034
Psychological characteristics			
Anxiety symptoms (HADS-A)	10.44 ± 0.73	9.95 ± 1.46	0.026
Depressive symptoms (HADS-D)	9.58 ± 1.52	9.21 ± 1.88	0.250
Physical quality of life (WHOQOL-BREF)	18.33 ± 2.73	17.60 ± 4.36	0.287
Psychological quality of life (WHOQOL-BREF)	16.37 ± 2.70	16.18 ± 3.72	0.756
Illness coherence (IPQ-R)	3.76 ± 0.50	3.59 ± 0.57	0.093

Values are presented as mean ± standard deviation or *n* (%). *p* values refer to between-group comparisons using Welch independent-samples *t*-tests for continuous variables and Pearson’s χ^2^ or Fisher’s exact tests for categorical variables, as appropriate.

**Table 2:** Pearson correlations between the measures examined in the present study.

	1	2	3	4	5	6	7
1. Illness coherence (T1)	1						
2. Exercise (T1-T2)	−0.121						
3. Pain intensity (T3)	0.210 *	−0.726 ***					
4. Pain interference (T3)	0.210 *	−0.736 ***	0.982 ***				
5. Anxiety (T3)	0.211 *	−0.659 ***	0.892 ***	0.890 ***			
6. Depression (T3)	0.209 *	−0.657 ***	0.906 ***	0.899 ***	0.920 ***		
7. Physical QoL (T3)	−0.254 **	0.683 ***	−0.888 ***	−0.914 ***	−0.848 ***	−0.831 ***	
8. Psychological QoL (T3)	−0.183	0.704 ***	−0.863 ***	−0.897 ***	−0.841 ***	−0.840 ***	0.947 ***

* *p* < 0.05, ** *p* < 0.01, *** *p* < 0.001.

## Data Availability

The datasets generated and analyzed during the current study are not publicly available due to ethical and privacy restrictions but are available from the corresponding author upon reasonable request.

## Supplementary Materials

The following supporting information can be downloaded at https://www.mdpi.com/article/10.3390/healthcare14172843/s1. Figure S1: CONSORT flow diagram of participant recruitment, allocation, follow-up, and analysis; Figure S2: Moderated mediation model of the association between illness coherence and patient-reported outcomes through exercise adherence, by intervention group (exercise-only vs. combined exercise plus diaphragmatic breathing). Dashed paths denote non-significant associations. Lower panel reports the corresponding indices of moderated mediation. Covariates included in each model: age, education, gender.

## Abbreviations

The following abbreviations are used in this manuscript:

BPI-SF	Brief Pain Inventory–Short Form
CSM	Common-Sense Model of Self-Regulation
HADS	Hospital Anxiety and Depression Scale
HADS-A	Hospital Anxiety and Depression Scale–Anxiety subscale
HADS-D	Hospital Anxiety and Depression Scale–Depression subscale
IPQ-R	Revised Illness Perception Questionnaire
PROCESS	PROCESS macro for SPSS
QoL	Quality of Life
RCT	Randomized Controlled Trial
SPSS	Statistical Package for the Social Sciences
WHOQOL-BREF	World Health Organization Quality of Life Instrument, Brief Version
