# Supplementary material for: Illness Coherence, Exercise Adherence, and Patient-Reported Outcomes in Chronic Spinal Pain: A Moderated Mediation Analysis
Source: Healthcare (Basel). 2026 Sep 3;14(17):2843. doi: 10.3390/healthcare14172843 (PMC13565878; doi:10.3390/healthcare14172843)
Supplement: Supplementary file 1 [file healthcare-14-02843-s001.zip › healthcare-4470401-supplementary.pdf]

Supplementary Materials

Illness Coherence, Exercise Adherence, and Patient-Reported Outcomes in Chronic Spinal Pain: A Moderated Mediation Analysis

Kalliopi Vlastou, Anastasia Beneka, Evangelos Bebetsos, Maria Basta, Charidimos Tzagarakis, Evangelos Karademas, Izolde Bouloukaki, Dimitris Trivizadakis and Panagiotis Simos

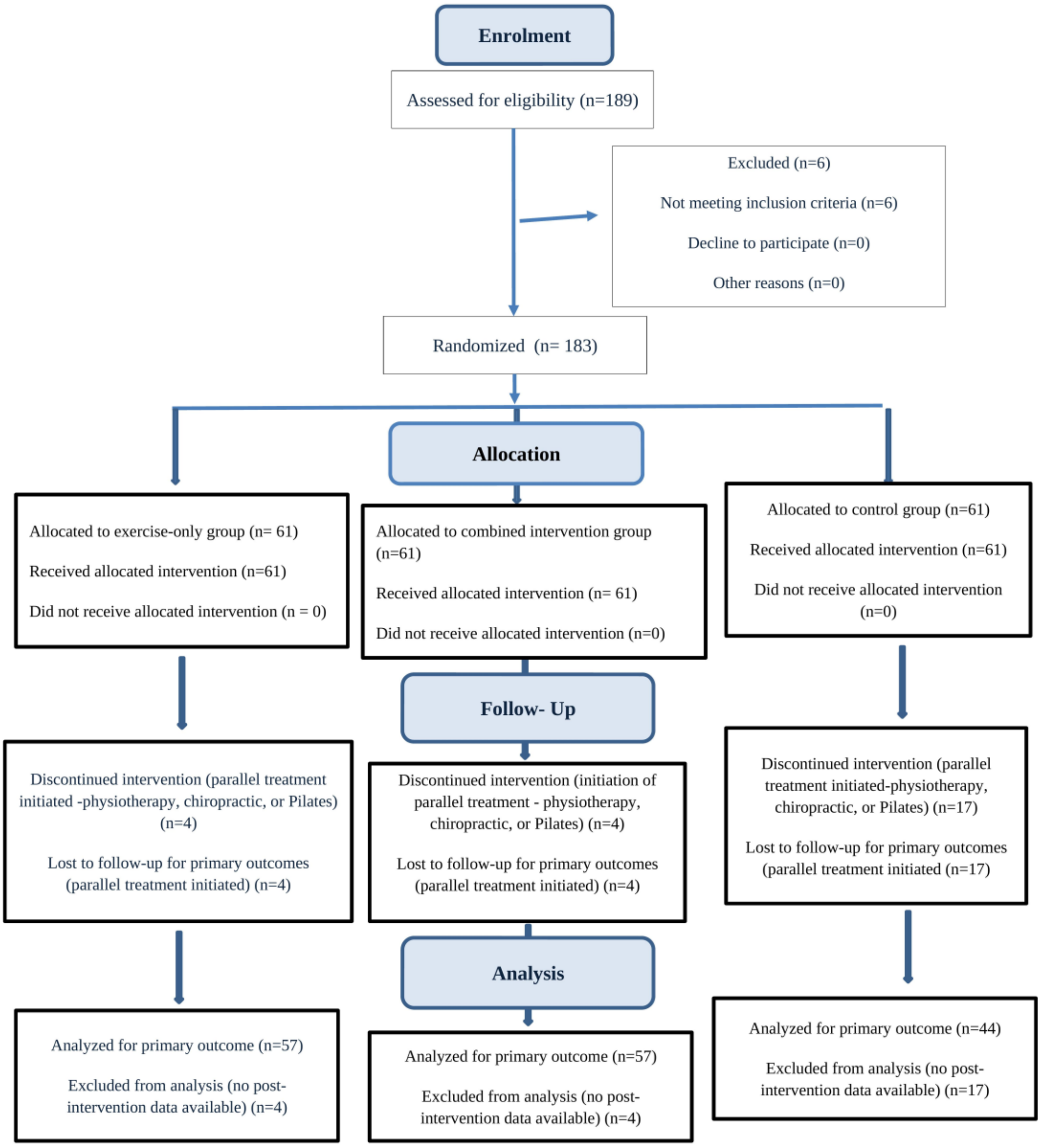

Figure S1. CONSORT flow diagram of participant recruitment, allocation, follow-up, and analysis.

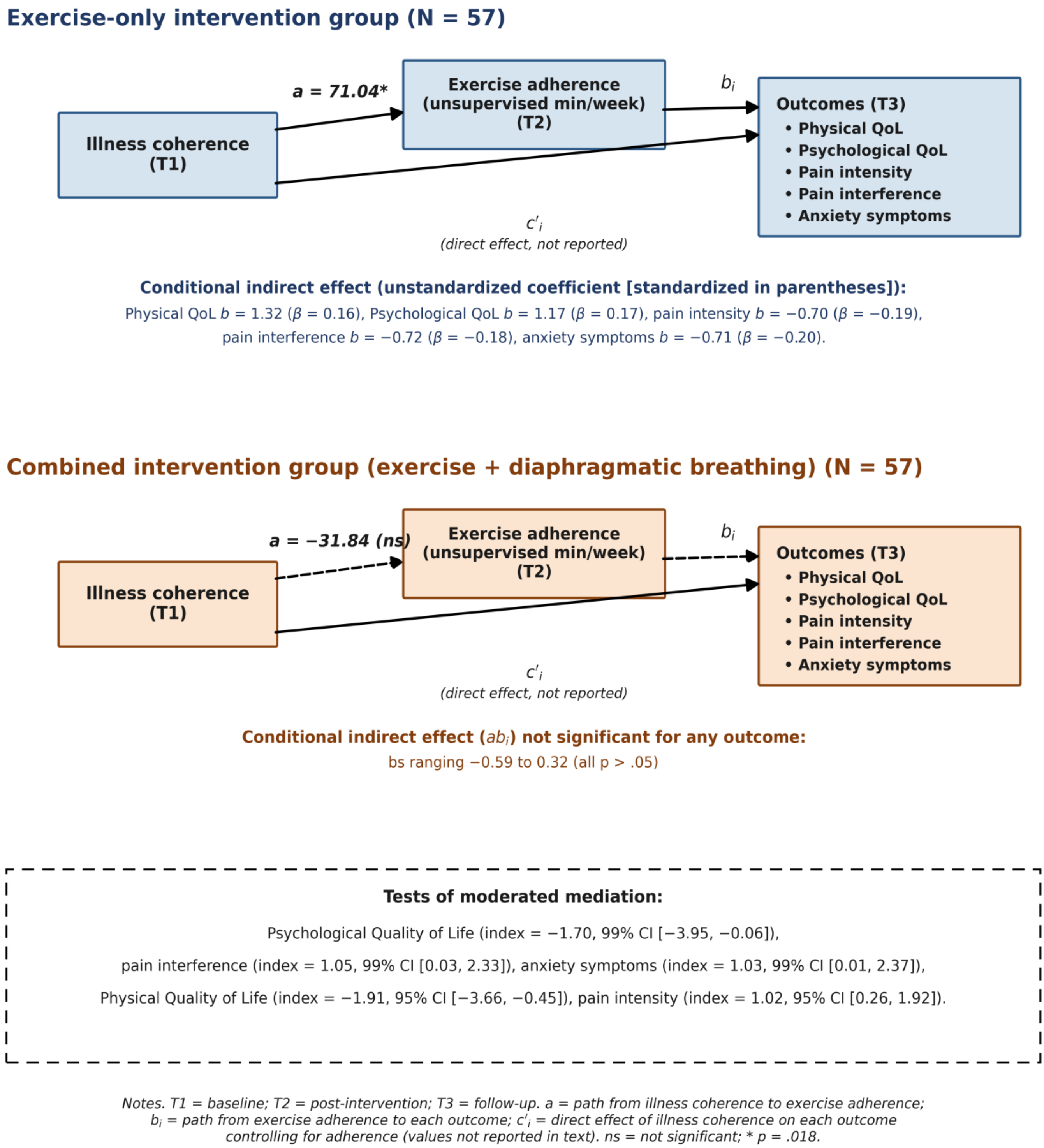

Figure S2. Moderated mediation model of the association between illness coherence and patient-reported outcomes through exercise adherence, by intervention group (exercise-only vs. combined exercise plus diaphragmatic breathing). Dashed paths denote non-significant associations. Lower panel reports the corresponding indices of moderated mediation. Covariates included in each model: age, education, gender.
